# Supplementary material for: The peripheral blood mononuclear cells preparation and the hematology of Varanus salvator
Source: PLoS One. 2022 Jul 22;17(7):e0269108. doi: 10.1371/journal.pone.0269108 (PMC9307208; doi:10.1371/journal.pone.0269108)
Supplement: Attachment — Submitted filename: Response to Reviewers_Varanus.docx [file pone.0269108.s001.docx]

Dear reviewers

According to some points raised by the academic reviewers. Please see the responses to each point below. Many thanks for all the helpful points.

Your sincerely

The authors of manuscript

The peripheral blood mononuclear cells preparation and the hematology of *Varanus salvator*

**Reviewer #1:**

The ms is aimed to evaluate the proper anticoagulants for the peripheral blood mononuclear cells (PBMCs) isolation and to analyse the effect of culture temperature on cell proliferation in the Varanus salvator’s. The ms is well written and the aims clearly explained and analysed. The main weakness I found rely with sample size and statistical analyses.

1) Males and females have been cumulated, but sex based differences are commonly found in lizards. So I suggest to separate sexes in all the analyes

**Response :** Unfortunately, We took blood sample from Varanus in nature, wildlife, which was quite aggressive. For PBMC experiment, we design to do the laboratory work on every sample on same day and same environment. Since, we do not have sex-based information, the new statistical analyzes were done and explained as non-parametric statistic in the manuscript revision.

2) Statistical methods are very poorly described, and in some cases are not fully appropriated For example, The simple ANOVA is not appropriate when comparing means with repeated sampling wihin individuals as, for example, in the analysis of the effect of temperature on cell proliferation (see Sacchi R, Mangiacotti M, Scali S, Coladonato AJ, Pitoni S, Falaschi M, et al. (2020) Statistical methodology for the evaluation of leukocyte data in wild reptile populations: A case study with the common wall lizard (Podarcis muralis). PLoS ONE 15(8): e0237992.). The data reported in figg 1 and fig 2 should be statistically processed by a LMM in which the number of cells is the dependent variable; the day, the treatments (temperature and anticoagulant) and their interactions are the predictors, while the individual is the random effect to account for repeated measuring.

**Response :** The statistical analyzes were revised, the non-parametric statistical analysis should be appropriated for this experimental design. We performed the new statistical analysis which you can see in the revision version of manuscript. In an unmarked version of revised paper, “Statistical analysis” was edited and explained clearer (Line: 143-164) which made much revision along the “Results and discussion part”, especially the discussion of PBMC experiment (start from line 166).

3)Further, body size has been totally omitted in the analysis, but we know that size (i.e. age) could affect cell growth.

**Response:** Unfortunately, as we mention previously in 1), however, we try to handle the Varanus that not much vary in size and not smaller that what we mention in “Materials and methods: Animals and sampling” part. The new statistical analysis might moderate this factor.

4) I believe that some of the differences between this and previous studies pointed out in several part of the ms might be due to the not appropriate statistical treatment of data

**Response:** We do agree and concern. We had revised all the statistic. The manuscript was expanded with 6 Tables and 2 figures. Thank you very much for your advice. After statistical revision, it provided a precious information with additional explanation and met the objective of the manuscript.

5)A further problem rely with the reference intervals, as reported in table 1. What do errors represent? I suppose standard deviations, but 95% CI intervals shoud be more appropriate. (see Dickinson VM, Jarchow JL, Trueblood MH. Hematology and plasma biochemistry reference range values for free-ranging desert tortoises in Arizona. J Wildl Dis. 2002; 38:143–153.)

**Response:** The hematological part was revised and reported by median, standard error and 95% confidence interval. “Results and discussion” were edited. See “Table 5 and 6”.

A final point is the ripercussion in conservation of the results of this study (line 101). Since only captive individuals have been considered, reference interval and physiological responses here analysed cannot directly extrapolated to wild populations, since captivity has relevant effects on blood parameters. In wild populations the variability of blood values is much higher than that in captive individulas (see Sacchi R, Mangiacotti M, Scali S, Coladonato AJ, Pitoni S, Falaschi M, et al. (2020) Statistical methodology for the evaluation of leukocyte data in wild reptile populations: A case study with the common wall lizard (Podarcis muralis). PLoS ONE 15(8): e0237992.). I think this point should be accounted when discussing the relevance of the results of the study. I think that main ripercussion of the results concenrca pets, captive animals and veterinary procedures, rather than species conservation

**Response:** We would like to confirm that the samples were took from wild reptile population. In our country, *Varanus salvator* is wild animal which under the wildlife protection according to the list of Thailand protected species. It's illegal if people feed or breed them. So, this study was authorized by the department of national park wildlife and plant conservation. Those Varanus just were protected and taken care by Khao-son Wildlife Breeding station, however, no one breed or feed them. They live in nature as wild animals.

**Reviewer #2:**

The immunological characteristics of the Varanus Salvator are very unknown. To evaluate the hematological characteristics and discriminate some practical conditions to culture, immune cells (PBMCs) of Varanus Salvator have an interest and it is a good tool to study. The descriptive study is well executed and presented.

Some questions have to be resolved:

Material and Methods

Lines 118-119. All blood samples, both the obtained with heparin and the obtained with EDTA were treated with heparin? Can you study the effects of EDTA if all samples were treated with heparin?

**Response:** One objective is to evaluate the proper anticoagulants for the collecting for the PBMCs isolation. Previously, we were interested in what kind of anticoagulant that proper to preserve and transfer to the laboratory. The laboratory has standard protocol of washing reagent preparation. Thank you very much for your notice that also pushes my mind, however, washing process went fast then follow with no anticoagulant in culture medium.

Lines 127-128. You indicate that the PBMCs lifespan were monitored at day 1, 2, 3, 4, 5, 6, 7, 8, 9, and 10 were by Trypan blue method; however, in the figures you indicate the cell number at 0 days. How you determine the cell counts at 0 days? Perhaps the high increase in cells at day 1 respect to day 0 could be results of different measurement techniques of cell count these days.

**Response:** We would like to confirm that we used the same counting method, Trypan blue.

Results and Discussion

Can you explain the proliferation of PBMCs in culture? PBMCs increase about three times the first culture day. Can PBMCs proliferate in culture?

**Response:** Two explanations are in the revision version of manuscript, Discussion: Line 225 - 235.
